# *SituSeq*: an offline protocol for rapid and remote Nanopore 16S rRNA amplicon sequence analysis

**DOI:** 10.1038/s43705-023-00239-3

**Published:** 2023-04-20

**Authors:** Jackie Zorz, Carmen Li, Anirban Chakraborty, Daniel A. Gittins, Taylor Surcon, Natasha Morrison, Robbie Bennett, Adam MacDonald, Casey R. J. Hubert

**Affiliations:** 1 Department of Biological Sciences, University of Calgary, Calgary, AB Canada; 2 Department of Biological Sciences, Idaho State University, Pocatello, ID USA; 3 Department of Natural Resources and Renewables, Government of Nova Scotia, Halifax, NS Canada; 4 Natural Resources Canada, Geological Survey of Canada-Atlantic, Dartmouth, NS Canada

**Keywords:** Environmental microbiology, DNA sequencing, Microbial ecology, Infectious-disease diagnostics, Microbiome

## Abstract

Microbiome analysis through 16S rRNA gene sequencing is a crucial tool for understanding the microbial ecology of any habitat or ecosystem. However, workflows require large equipment, stable internet, and extensive computing power such that most of the work is performed far away from sample collection in both space and time. Performing amplicon sequencing and analysis at sample collection would have positive implications in many instances including remote fieldwork and point-of-care medical diagnoses. Here we present *SituSeq*, an offline and portable workflow for the sequencing and analysis of 16S rRNA gene amplicons using Nanopore sequencing and a standard laptop computer. *SituSeq* was validated by comparing Nanopore 16S rRNA gene amplicons, Illumina 16S rRNA gene amplicons, and Illumina metagenomes, sequenced using the same environmental DNA. Comparisons revealed consistent community composition, ecological trends, and sequence identity across platforms. Correlation between the abundance of taxa in each taxonomic level in Illumina and Nanopore data sets was high (Pearson’s *r* > 0.9), and over 70% of Illumina 16S rRNA gene sequences matched a Nanopore sequence with greater than 97% sequence identity. On board a research vessel on the open ocean, *SituSeq* was used to analyze amplicon sequences from deep sea sediments less than 2 h after sequencing, and 8 h after sample collection. The rapidly available results informed decisions about subsequent sampling in near real-time while the offshore expedition was still underway. *SituSeq* is a portable and user-friendly workflow that helps to bring the power of microbial genomics and diagnostics to many more researchers and situations.

## Introduction

Examining the microbiome of extreme and remote environments has increased our collective understanding of microbial physiology and diversity [1, 2]. Collecting samples from these remote locations requires fieldwork that can be expensive and time consuming. Fieldwork is also logistically challenging as it can be complex to move people, equipment, and samples long distances, across borders, and through difficult terrain. In these situations, every sample taken is valuable in terms of the resources required for collection. Despite this, it is not always certain that the samples will address the research question, sometimes leaving researchers in situations where they are uninformed during sampling campaigns.

Sequencing of microbial genes (e.g., the 16S rRNA gene) is often used for environmental monitoring and medical diagnostics, as well as to identify indicators of environmental conditions, disturbances, and diseases [3, 4]. Rapid analysis of 16S rRNA gene diversity in a microbial community could be used to quickly characterize a sample in circumstances when time or access to resources are limited. Sequencing with Oxford Nanopore technology, or “third generation” sequencing, is quickly gaining favor in the microbiological research community [5–8]. Nanopore sequencing allows for the continuous sequencing of long sequences of nucleotides, e.g., the full-length, 1500 bp 16S rRNA gene, enabling better taxonomic resolution than much shorter Illumina sequences [9]. While several Nanopore platforms have been developed and are in use, the MinION sequencer has gained recognition as relatively inexpensive and exceptionally portable. To exemplify its portability, the MinION sequencer has been used to sequence DNA in space [10–12], in remote field locations like the high Arctic [13] and during a ski touring expedition in Iceland [14]. In most of these previous cases of *in situ* sequencing, researchers have had to wait to analyze data until there was an internet connection or sufficient computing power available. This delay prevents the collection of meaningful information about field samples until much later, when field trips have ended and opportunities to adjust sampling strategies are gone. It would be advantageous to retrieve DNA sequence data in real time in the field, thereby informing decisions about whether to prioritize a site or move on to other sampling opportunities with the limited time and resources available [5].

A drawback of Nanopore technology has been its low accuracy compared to conventional “second generation” sequencing-by-synthesis technologies like Illumina [15, 16]. However, the accuracy of Nanopore sequencing is rapidly improving, with current estimates of >95% for raw read accuracy, leading to >99.99% consensus accuracies for both metagenomics and amplicon sequencing [17, 18]. Furthermore, recent chemistry updates (R10.4) report 99% read accuracy and have enabled near-finished genomes without the need for polishing with short read Illumina sequences [18]. Nanopore sequencing is quickly approaching parity with other sequencing platforms in this regard [19–21].

Here, we present an amplicon sequencing workflow, *SituSeq*, designed to be used remotely with Nanopore sequencing. The workflow uses a MinION sequencer with the inexpensive Flongle adapter, and a completely offline bioinformatics analysis pipeline with a pre-loaded database. The *SituSeq* method can be completed in less than an hour, and the entire process from DNA extraction to data visualization can be completed in less than 8 h. The workflow was tested during remote fieldwork in the NW Atlantic Ocean, assessing freshly collected deep sea sediments (>2000 m water column) ~300 km offshore of Nova Scotia, Canada. Subsurface marine sediments contain a large amount of microbial biomass [21, 22] yet harbor many uncultured and understudied taxa [23–25]. This is in large part due to the requirement for expensive resources (including ships for coring, personnel, and equipment), to collect samples in an often-limited time frame. These constraints make deep sea sediment sampling an ideal setting to test the implementation of this *in situ* sequencing method. Sequencing and analysis were carried out while at sea without internet connection and, in turn, informed subsequent sample collection during the remainder of the expedition. Refinement of the workflow was achieved using the same protocols back in the laboratory, and by comparing results to standard sequencing of amplicons and metagenomes from the same DNA using Illumina technologies. The code for running this analysis is available at https://github.com/jkzorz/SituSeq and in Data S1.

## Materials and methods

For the initial test of this protocol, DNA extraction, PCR, Nanopore library preparation, Nanopore sequencing, and subsequent data analysis were conducted at sea aboard the R/V *Atlantic Condor* in August 2021 [26]. This investigational effort resulted in the sequencing and analysis of deep sea sediment samples within hours of their retrieval, allowing the microbial community in each sample—including the presence of hydrocarbon seep ‘indicator’ lineages [27] (Data S2)—to be assessed. Back in the laboratory, we then compared the results of the *SituSeq* Nanopore method conducted on 40 samples to the results of standard Illumina sequencing of 16S rRNA gene amplicons and metagenomes. Sequencing on all platforms was performed using the same extracted DNA. Below are the details of the *SituSeq* workflow and the comparison to Illumina sequencing.

### Sample collection and description

**Fig. 1:**
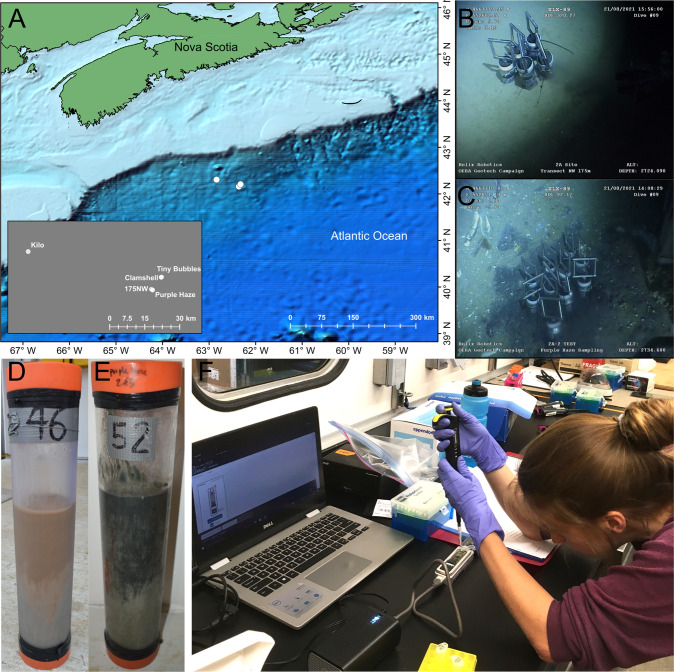
Research expedition on the Scotian slope offshore Nova Scotia. **A** Sites sampled in the Northwest Atlantic Ocean. **B** ROV footage from the background site “175NW”. **C** ROV footage from the hydrocarbon seep site “Purple Haze”. **D** Image of the 31 cm long core taken from site “175NW”. **E** Image of the 36 cm long core taken from site “Purple Haze”. **F** A Nanopore MinION sequencing run performed while at sea during the sampling expedition.

This study examined 40 marine sediment samples collected from different depth intervals within five push cores, each sampling approximately the top 30–40 cm of the seabed. Push coring used a remotely operated vehicle (ROV; Helix Robotics), deployed from the R/V *Atlantic Condor*. Names of the five push coring sites are: “Purple Haze”, “Tiny Bubbles”, “Kilo”, “Clamshell”, and “175NW” (Fig. 1A). The first four sites showed visual evidence of hydrocarbon seepage and/or macroscopic fauna (e.g., shells at Purple Haze are indicative of chemosynthetic biological activity; Fig. 1C, E), whereas “175NW” lacked distinguishable features, and appeared similar to the surrounding abyssal sea floor (Fig. 1B, D). The entire push core was sectioned on board into 4 cm long intervals that were stored immediately at −80 °C.

### Library preparation and sequencing methods

#### DNA extraction

DNA was extracted from the 40 marine sediment samples using the DNeasy PowerLyzer PowerSoil Kit (Qiagen, Germany*)* per the manufacturer’s instructions. To increase DNA yield, between 0.5–1 g of sediment was added to each lysis bead tube. Two 45 s rounds of bead beating using an Omni Bead Ruptor 24 bead beater (Omni-Inc, USA) at speed 5 were used to lyse cells. DNA was eluted in 70 µL of elution buffer (C6) following a 2 min room temperature incubation. DNA concentration was measured with a Qubit fluorometric assay (ThermoFisher, USA). Not enough DNA was retrieved from the 12–16 cm depth sample of site 175NW and so it was not included in the 40 samples used for sequencing and subsequent analyses.

#### Full length 16S rRNA gene barcoding PCR for Nanopore sequencing

Amplification of the full length 16S rRNA gene, clean up, and library preparation were performed using the 16S Barcoding Kit (SQK-RAB204, Oxford Nanopore Technologies, UK) per manufacturer’s instructions with minor modifications. This kit contains primers 27 F/1492 R for amplification of the full-length 16S rRNA gene (Table S1), and has 12 barcoded primer pairs, allowing for the simultaneous sequencing of 12 samples. Instead of LongAmp Taq 2x master mix, KAPA HiFi HotStart master mix (Roche, Switzerland) was used to remain consistent with the standard Illumina 16S rRNA PCR protocol [27]. As per kit instructions, 10 ng of DNA per sample was used as template for the PCR except in samples with low extracted DNA concentrations (<1 ng/uL, determined using a Qubit fluorometer, ThermoFisher, USA) where at least 5 ng of template DNA was used. The PCR cycling conditions were altered slightly to accommodate the different polymerase enzyme and to improve extension conditions [28]. Alterations included longer denaturation and annealing phases (30 s and 45 s, respectively, in the cycles), and a higher temperature for the extension (increase from 65 °C to 72 °C). The thermocycler (Mastercycler GSX1, Eppendorf, Germany) program used can be found in Table S2.

A difficulty encountered during the barcoding and sequencing process was that barcode 8 and barcode 10 of the 16S Barcoding Kit (batch no. SE04.10.0020) consistently resulted in low-yield PCR products insufficient for downstream analysis. To remedy this, samples originally amplified with barcode 8 and barcode 10 were re-amplified with other barcodes to obtain enough material for the remainder of the protocol. Therefore, it is recommended that all barcoded primers are tested with positive controls prior to use.

PCR products were purified with AMPure XP beads (Beckman Coulter, USA), according to the instructions in the 16S Barcoding Kit. After PCR clean-up, the Qubit fluorometric assay was used to quantify DNA prior to pooling and normalizing libraries. A blank DNA extraction and PCR was performed in the same manner as the samples, yielding DNA concentrations that were too low to be sequenced. Pooling of samples was done so that between 50–100 ng of total DNA was loaded in total, and between five and ten samples were included in each sequencing run. DNA was prepared for loading onto the Flongle adapter according to the Nanopore instructions. DNA prepared in this way from the 40 different samples were run in batches that spanned six separate sequencing runs.

#### Nanopore sequencing

Sequencing was conducted using the MinION with a Flongle flow cell (R9.4.1) and with a MinIT (MNT-001) (Oxford Nanopore Technologies, UK) for basecalling. At the time that this field expedition took place (summer 2021), the MinIT was available as a companion to the MinION but has since been discontinued. It can be replaced with a laptop or the Mk1b device for basecalling. The Flongle flow cell provided an adequate sequencing depth for 16S rRNA gene amplicon surveys at a very low cost (~$90 USD). Sequences were locally basecalled using MinKNOW (v 4.3.20) (Oxford Nanopore Technologies, UK), connected to a Dell Inspiron 13–7378 laptop with 16 GB RAM and 512 GB SSD (Dell, USA). The length of the sequencing runs was variable and depended on Flongle flow cell quality and desired number of sequences per sample. In general, runs were continued until the active pores in the flow cells were depleted. As an example, to obtain 5000 sequences per sample with six barcoded samples (i.e., 30,000 sequences total), using a Flongle with around half of the pores available (~60 pores), approximately 2 h of sequencing is required. On average, Nanopore amplicon libraries in this study contained 18,630 reads.

#### PCR amplification of V4 region of 16S rRNA gene and sequencing on an Illumina MiSeq

The same extracted DNA that was used for long-read Nanopore sequencing was used for short-read Illumina sequencing. Sample preparation and Illumina sequencing of the 40 samples was performed as previously described [29]. Briefly, the V4 region of the 16S rRNA gene was amplified using the 515 F/806 R universal primer set (Table S1) [30, 31]. The thermocycler (Mastercycler GSX1, Eppendorf, Germany) programs used can be found in Table S2. A blank DNA extraction and PCR was performed but yielded DNA that was too low to be included in a sequencing run. Amplicon samples were sequenced using Illumina’s v3 600-cycle (paired-end) reagent kit on an Illumina MiSeq benchtop sequencer (Illumina, USA). On average, Illumina amplicon libraries in this study contained 22,814 reads.

#### Metagenome sequencing

To verify taxonomic community compositions, shotgun metagenomes were sequenced from nine out of the 40 samples using an Illumina NovaSeq (Illumina, USA). The nine samples chosen for metagenomes were from multiple depths and included cores taken from areas with visual evidence of hydrocarbon seepage, and areas without. Libraries were prepared using a NEBNext Ultra II fragment library preparation kit (New England Biolabs, USA) with Covaris shearing (Covaris, USA). Libraries were then sequenced on a NovaSeq S4, 300 cycle run at the Center for Health Genomics and Informatics (University of Calgary, Calgary, Canada), producing approximately 100 M reads per sample.

### Data analysis

#### Nanopore analysis workflow – *SituSeq*

**Fig. 2:**
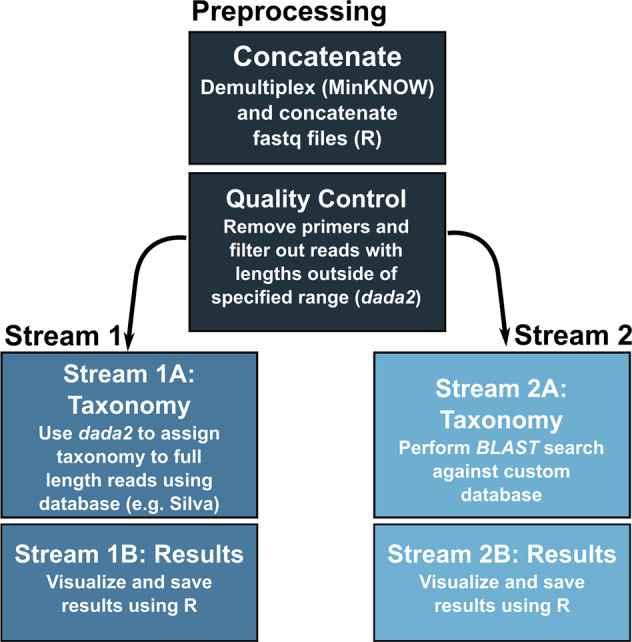
*SituSeq* bioinformatics workflow. The preprocessing script contains quality control steps that remove primers and filter sequences outside the specified length parameters. The Stream 1 workflow uses the *assignTaxonomy* function from *dada2* to assign taxonomy to all Nanopore sequences. The Stream 2 workflow performs a *BLASTn* search of the Nanopore sequences as queries against a custom database.

All code used for the analysis and instructions for analyzing data remotely and offline can be found at github.com/jkzorz/SituSeq. All software (R version > 4.2), packages (*dada2, ShortRead, tidyverse*, and *rBLAST*) [32–34], and databases used in the *SituSeq* workflow require an internet connection to install, but once installed, can be run offline on a standard laptop (e.g., a Dell Inspiron 13–7378 laptop with 16 GB RAM and 512 GB SSD). Only reads that passed the default Nanopore quality threshold (>Q7) were included in the analysis. An initial preprocessing script filters and trims reads, and then two analysis streams are offered: (1) taxonomic identification of all sequences with *dada2* [32] (program requirements: R), and (2) query of sequences against a pre-defined database of 16S rRNA gene sequences from species of interest using *BLASTn* (program requirements: R). Both methods are described below (Fig. 2).

The preprocessing script concatenates separate sequence files from the same sample, and then uses the *filterAndTrim* command from *dada2* to remove primers and sequences longer or shorter than expected. For the present analysis, the first and last 100 bp of sequence were trimmed to remove primers and barcodes (trimLeft and trimRight), and then sequence reads were filtered using minLen = 1200 and maxLen = 1800 (filtering and trimming parameters are adjustable).

The first analysis method, Stream 1, is conducted entirely in R. It involves Stream 1A for the taxonomic assignment of full-length 16S rRNA gene sequences using a locally downloaded database (e.g., the Silva 138.1 database, 134 Mb) [35] and the *assignTaxonomy* command from the program *dada2* (v1.20.0). Stream 1A includes an option, “subsample_depth”, that specifies the number of reads that libraries will be subsampled to. Increasing the subsample depth will increase identification of rare taxa but requires longer analysis time. Stringency of taxonomic assignment can be set through the “minBoot” parameter in Stream 1A, which refers to the minimum bootstrapping support required to return a taxonomic classification with *assignTaxonomy*. Stream 1B summarizes and visualizes the results from Stream 1A and allows the user to choose a taxonomic level for summary. For the present study, analyses were conducted on samples without subsampling sequences, and after subsampling all samples to 1000 sequences.

The second method, Stream 2, involves a *BLASTn* identity search (v2.12.0+), using the R package *rBLAST* [34], to interrogate library reads against a pre-defined database of 16S rRNA gene sequences belonging to species of interest for the application in question. In this particular instance, the database included indicator sequences from hydrocarbon seep-associated bacteria identified in this study area [27] and in cold seep sediments in the eastern Gulf of Mexico [29] (Data S2). The search database could include sequences from any number of species of interest to identify their presence in the samples being analyzed. The *BLASTn* command used required >97% identity, and the matches were filtered to remove any match with an e-value greater than 0. The parameters “perc_identity”, “alignment_length”, and “e_value”, are customizable using *SituSeq* Stream 2B. Because the *BLAST* search is relatively fast, Stream 2 does not include an option for subsampling.

#### Illumina analysis workflow

Samples sequenced on the Illumina MiSeq were analyzed using the *dada2* package in R [32] following its accompanying tutorial (https://benjjneb.github.io/dada2/tutorial.html). Only reads with a quality score >Q30 were included in the analysis. The samples were sequenced across two different MiSeq sequencing runs, such that the *learnErrors* and *dada* commands needed to be performed on each run separately. Two resulting ASV tables were then merged prior to taxonomic classification with the *mergeSequenceTables* command. Archaeal sequences were removed from Illumina libraries before comparison with the Nanopore libraries that were generated using bacteria-specific 16S rRNA gene primers. The Silva 138.1 database was again used for taxonomic assignment, in the same manner as the Nanopore analysis. All code used in the analysis of the Illumina amplicon data is provided at github.com**/**jkzorz**/**SituSeq.

#### Reconstruction of 16S rRNA genes from metagenomes

Illumina metagenome sequences underwent quality control using *bbduk* (BBTools suite; http://jgi.doe.gov/data-and-tools/bbtools), to remove the last base, adapters, contaminants, and low quality sequences. *PhyloFlash* v3.4 [36] was then used with the parameters: *-poscov -treemap -log -readlength 150*, to assemble and extract 16S rRNA sequences from the reads, and to assign taxonomy to those sequences using the Silva 138.1 database. Archaea and Eukaryote sequences were removed before calculating relative abundances of bacterial taxa to compare with the Nanopore libraries that were generated using bacteria-specific 16S rRNA gene primers. From the *phyloFlash* output, the files named LIBNAME.phyloFlash.NTUabundance.csv were used to calculate the relative abundance of taxa, and the files named LIBNAME.all.final.fasta, containing all assembled and reconstructed 16S rRNA gene sequences, were used for *BLAST* searches against Nanopore amplicon sequences.

#### Comparison of Illumina and Nanopore sequences

Taxonomic classifications and relative abundances of sequences were used to compare Illumina and Nanopore sequencing of the 16S rRNA gene from the same 40 samples. A three-way comparison between Illumina MiSeq amplicons, Nanopore amplicons, and *phyloFlash* sequences from Illumina metagenomes, was conducted for the nine samples that also had metagenomes. Many species found in deep sea sediments are poorly classified at finer taxonomic resolution, therefore the phylum level was used for the main comparisons, while comparisons at finer taxonomic levels between the Nanopore amplicons and the Illumina amplicons are included in Figs. S1–S3. The relative abundances of phyla, classes, families, orders, genera, and phylotypes (highest classified taxonomy) were calculated using each sequencing technology and compared to assess any potential biases within the protocol. NMDS ordinations, ANOSIM tests, and Mantel tests were performed in R using the *vegan* package (v. 2.6-2) [37]. Bray-Curtis dissimilarity was used as the dissimilarity measure for NMDS ordinations as well as the ANOSIM and Mantel tests. Differentially abundant phyla and genera were identified based on a biserial correlation calculated using the *multipatt* function in the *indicspecies* package (v. 1.7.7) [38] in R. Differentially abundant phyla and genera in different seabed locations were identified by grouping data separately for Illumina and Nanopore data sets. Combined Nanopore and Illumina data sets were used when identifying differentially abundant phyla based on sequencing technology. Pearson correlation between the relative abundance of taxa in the Illumina, Nanopore, and *phyloFlash* data sets was calculated using the *cor* function in R.

*BLAST* searches were done to directly compare sequence identities in a pair-wise manner for the three sequencing strategies (Nanopore 16S rRNA gene amplicons, Illumina 16S rRNA gene amplicons, and Illumina metagenomes). A custom searchable database was created from the Nanopore sequences using the command *makeblastdb*. *BLASTn* searches were performed using the Illumina MiSeq and the *phyloFlash* metagenome sequences as queries. For the Illumina MiSeq searches, a requirement of 97% identity and a match longer than 230 bp was needed to be counted as a match. A second *BLASTn* search was done to search for 100% similarity between the Illumina MiSeq and Nanopore sequences. An unlimited (1000) amount of target sequence matches were included to allow for short read amplicons to match multiple Nanopore sequences. A *BLASTn* search with the parameters of 97% identity over 800 bp was used to query the reconstructed *phyloFlash* 16S rRNA gene sequences against the Nanopore sequence database.

## Results

### Illumina MiSeq 16S rRNA gene sequencing validation of Nanopore results

#### Community composition is similar regardless of amplicon sequencing method

After filtering for length, amplicon libraries sequenced with Nanopore had an average of 18,630 reads (maximum: 38,121; minimum: 1153). There were 745,171 full length Nanopore sequences retrieved in total (average length: 1403 bp after trimming and filtering). Illumina MiSeq amplicon sequencing resulted in a total of 912,570 bacterial sequences with an average of 22,814 bacterial reads per library (maximum: 84,462; minimum: 6819), and an average length of 253 bp. In total, 7272 unique bacterial ASVs were formed from the Illumina reads.

In total, 66 phyla were identified in the Nanopore data set, and 65 phyla were identified in the Illumina data set. Four phyla (Apal-E12, FW113, MAT-CR-M4-B07, and Synergistota) were only present in the Nanopore data set, while three phyla (Deinococcota, Halanaerobiaeota, and Poribacteria) were only present in the Illumina data set. Halanaerobiaeota, Poribacteria, and MAT-CR-M4-B07 were identified as differentially abundant between the methods as discussed below and shown in Table S3, whereas the other four phyla were not recognized in the comparative analysis likely due to very low abundance.

**Fig. 3:**
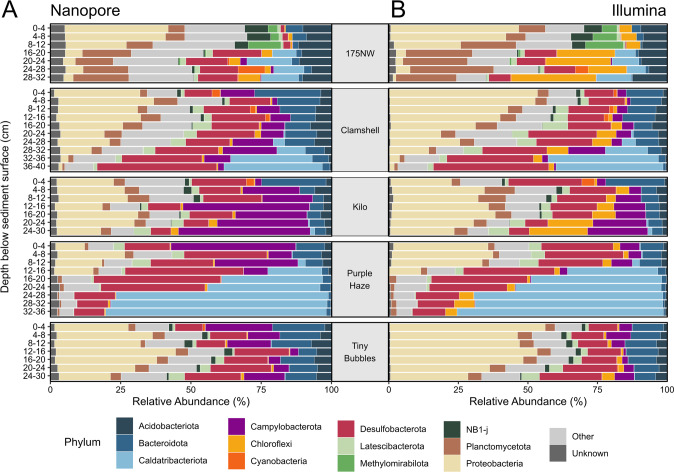
Relative abundance of most abundant phyla. **A** Abundance of phyla across samples from Nanopore data set. **B** Abundance of phyla across samples from Illumina data set. “Unknown” represents the sequences not identified at the phylum level and “Other” represents the less abundant phyla.

The most abundant phyla on average across all 40 samples from the Nanopore data set were Proteobacteria (18.7%), Desulfobacterota (17.7%), Caldatribacteriota (11.5%), Campylobacterota (11.4%), and Bacteroidota (7.7%) (Fig. 3A). The most abundant groups at the genus level using Nanopore were unclassified (65%), *Sulfurovum* (7.4%), SEEP-SRB1 (5.3%), and *Sulfurimonas* (3.0%). Illumina results for the same samples grouped at the phylum level were similar with the most abundant being Proteobacteria (26.0%), Desulfobacterota (16.7%), Caldatribacteriota (12.1%), Planctomycetota (6.9%), and Bacteroidota (6.1%) (Fig. 3B). The most abundant groups at the genus level using Illumina were unclassified (65%), SEEP-SRB1 (7.8%), *Sulfurovum* (2.7%), and Marine Methylotrophic Group 2 (2.1%). Observing these taxa is consistent with the microbiome previously reported for deep sea sediments of the Scotian Shelf [27, 39]. Within the Nanopore data set, 2.9 ± 1.2% of the community had no taxonomic classification at the phylum level, compared to 0.8% ± 0.7% of the community within the Illumina data set. However, at the genus level, there were fewer sequences without classification within the long-read Nanopore data set (65 ± 12% of sequences and relative abundance) than in the short-read Illumina data set (80% of ASV sequences, collectively comprising 65% ± 11% relative abundance of the Illumina data set). While the longer Nanopore 16S rRNA gene amplicons likely improve taxonomic assignment at finer resolution compared to the shorter Illumina amplicons [9], both of these high proportions also reflect the under-studied nature of deep sea sediment ecosystems [23–25], resulting in their microbiomes not being well represented or resolved in taxonomic databases.

**Fig. 4:**
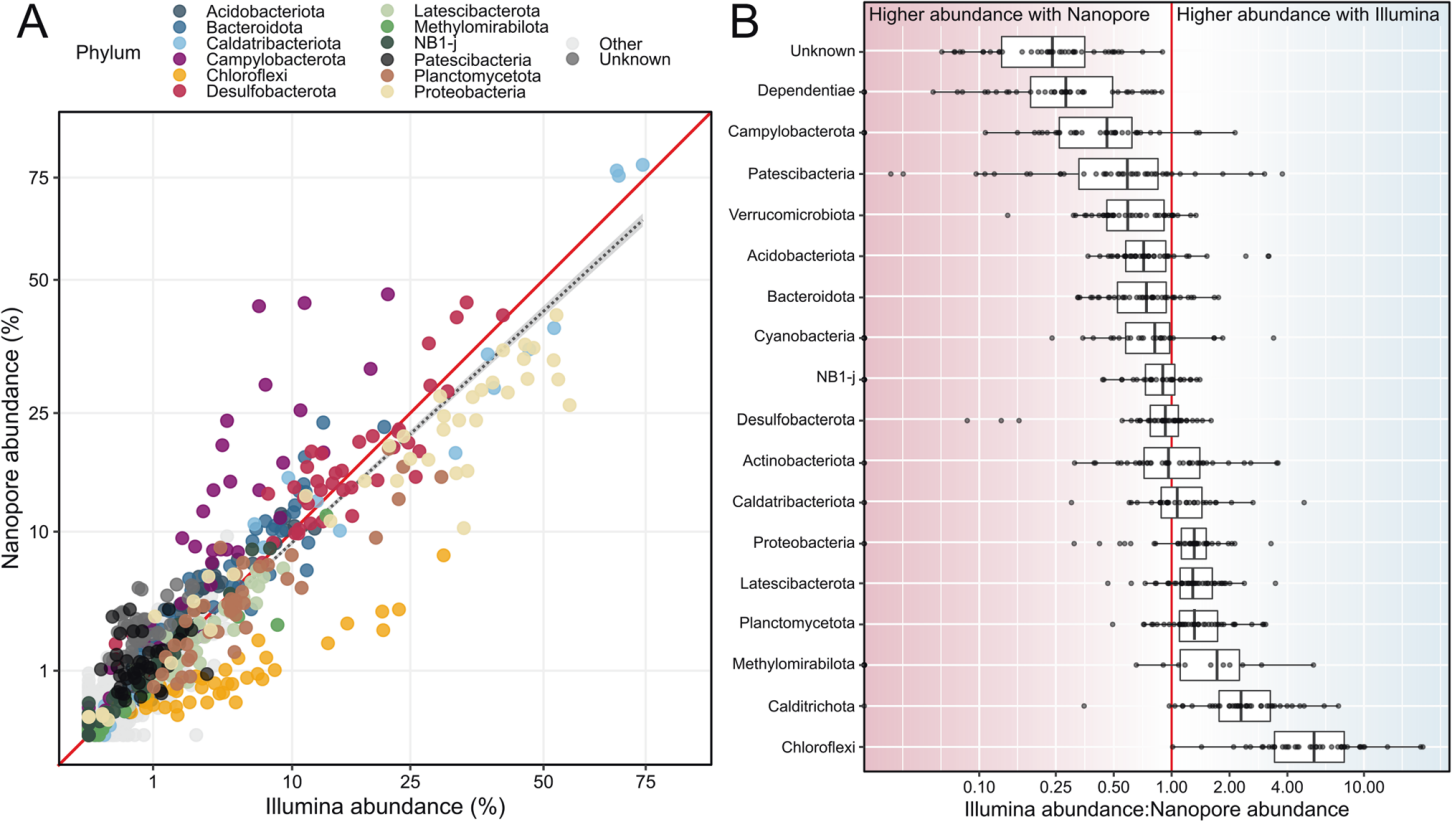
Comparison of Nanopore and Illumina amplicon sequencing. **A** Correlation between abundance of phyla sequenced with Nanopore (*y*-axis), and Illumina (x-axis) technology (Pearson’s *r* = 0.905). Abundant phyla are colored, “Unknown” represents the sequences not identified at the phylum level, and “Other” represents the less abundant phyla. The black dashed line shows the linear relationship between Nanopore and Illumina abundances, and the red line shows a 1:1 ratio. The axes have been square root transformed. **B** The ratio of Illumina abundance to Nanopore abundance of select phyla. Ratios for individual samples are overlaid on the boxplots. The red line shows a 1:1 ratio. The same comparisons at other taxonomic levels can be found in Figs. S1–3.

Overall, there was high correlation between the relative abundance of phyla from Nanopore and Illumina data sets (Pearson’s *r* = 0.905) (Fig. 4A). There were, however, differences between the sequencing technologies in terms of the relative abundance of certain phyla. Chloroflexi had a much higher relative abundance (6.4x ± 4.2x higher) in samples sequenced with Illumina technology than in samples sequenced with Nanopore (Fig. 4B). The phylum Campylobacterota, in contrast, was more abundant in Nanopore samples (2.8x ± 1.8x) compared to Illumina samples. Table S3 contains the phyla that were differentially abundant between the sequencing methods.

Agreement between Nanopore and Illumina data sets was also examined at finer taxonomic resolution. Pearson correlation between Nanopore and Illumina relative abundances at the class, order, family, and genus levels were 0.902, 0.914, 0.958, and 0.976, respectively (Fig. S1). Because a large proportion of the sequences were unclassified at finer taxonomic levels in both Nanopore and Illumina data sets (rendering comparisons arbitrary), we also performed comparisons using the highest level of taxonomic classification that was assigned to a sequence, i.e., its “phylotype”. The Pearson correlation between phylotypes in the Nanopore and Illumina data sets was 0.885 (Fig. S2A). Discrepancies in relative abundances of taxa at different taxonomic levels were also evaluated (Figs S2B, S3). At the genus level, *Algorimarina* accounted for 2.6% of the Nanopore data set on average but was not identified in the Illumina data set (Fig. S3). Strong *BLAST* matches between Nanopore sequences assigned to *Algorimarina* and Illumina sequences showed that the corresponding Illumina sequences were being assigned the taxonomic classification of “SEEP-SRB1”. This suggests that the full-length Nanopore 16S rRNA gene sequences, compared to just the V4 region used for Illumina sequencing, provided additional information that allowed for differentiation between *Algorimarina* and SEEP-SRB1 taxa. Other discrepancies from rarer genera include Candidatus *Scalindua* (0.6% average abundance) and *Lutimonas* (0.2% average abundance), which were 40x and 32x more abundant in the Illumina data set compared to the Nanopore data set, respectively (Data S3 and S4).

#### Ecological trends are similar regardless of amplicon sequencing method

**Fig. 5:**
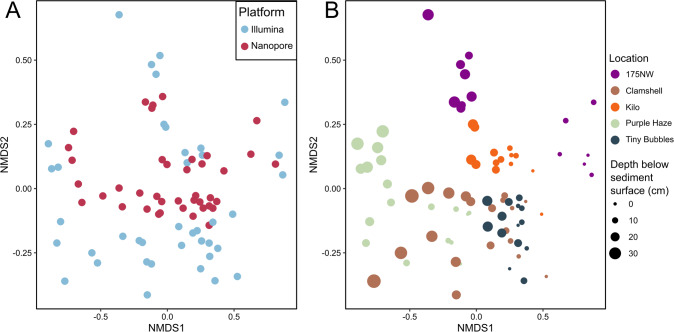
NMDS plots with combined Nanopore and Illumina data sets. The same NMDS plots based on Bray-Curtis dissimilarity are shown with samples (points) colored and sized based on different parameters. **A** Sample color indicates sequencing technology. **B** Samples colored on location, with size proportional to depth below the sediment surface. Stress = 0.1.

Nanopore and Illumina amplicon data sets were combined at the phylum level to determine the effects of sequencing technology (a combination of library preparation and sequencing platform) on broad ecological conclusions (Fig. 5). Sequencing technology had a significant effect on microbial community composition (ANOSIM *p* = 0.008), but the strength of this impact was small (ANOSIM statistic *R* = 0.06) and did not mask the effect of sampling location (site) (ANOSIM statistic *R* = 0.38, *p* < 1e^−4^). Differences in microbial communities between samples in the combined data set were significantly correlated with differences between sediment depth intervals in the seabed (Mantel statistic r: 0.33, *p* < 1e^−4^). The same tests were repeated on data sets combined at class, order, family, genus, and phylotype levels, showing very similar results, and confirming that observed ecological trends are consistent regardless of the taxonomic level or sequencing method used for analysis (Table S4). When Nanopore and Illumina data sets were evaluated separately, the effect size of location and correlation with depth were significant (*p* < 1e^−4^) and similar to the combined data sets. ANOSIM statistics of location for Nanopore-only and Illumina-only data sets were 0.36 and 0.37, respectively, and Mantel statistics for Nanopore-only and Illumina-only data sets were 0.30 and 0.35, respectively. Therefore, the major ecological trends in the data were very comparable despite differences in primers and sequencing platforms.

#### Sequence identities are high between amplicon sequencing methods

A *BLAST* search was performed using the Illumina ASV sequences as queries against a custom database composed of the Nanopore sequences. This was done to compare Nanopore and Illumina data sets at a finer resolution, in a taxonomy-free manner without the constraints of incomplete taxonomic classifications. This revealed that 5303 (73%) out of the 7272 Illumina bacterial ASVs had *BLAST* hits with greater than 97% percent identity among the Nanopore sequences. Of the top 1000 most abundant Illumina ASVs (comprising 83% of the Illumina data set relative abundance), only 20 ASVs did not match to any of the Nanopore full length sequences. This indicates that the most abundant species in the community were identified within the Nanopore data set with high sequence agreement.

A *BLAST* search identifying sequence matches with 100% identity between Nanopore and Illumina sequences was also conducted to evaluate perfect matches. Of the 7272 Illumina ASVs, 567 (7.8%) had *BLAST* hits with 100% identity to a full length Nanopore sequence. Some of these ASVs had 100% identity to multiple Nanopore full length sequences (i.e., owing to Nanopore sequences differing from each other in other areas of the 16S rRNA gene), such that 1249 (0.17%) of the full-length Nanopore sequences matched perfectly to ASVs from the Illumina data set. Of the top 21 most abundant Illumina ASVs, 20 had 100% identity matches to Nanopore sequences (the exception being ASV8 from Chloroflexi), and in general, the more abundant Illumina ASVs had a higher number of 100% identical hits to the Nanopore sequences. For example, ASV1 (Caldatribacteriota) and ASV2 (*Sulfurovum*), had 100% matches to 87 and 37 unique Nanopore sequences, respectively. This highlights the range of sequence diversity that is missed when using shorter variable regions and shows the potential of full length 16S rRNA gene sequencing for greater taxonomic resolution past what is possible with short-read amplicons.

### Shotgun metagenome taxonomy validates Nanopore results

**Fig. 6:**
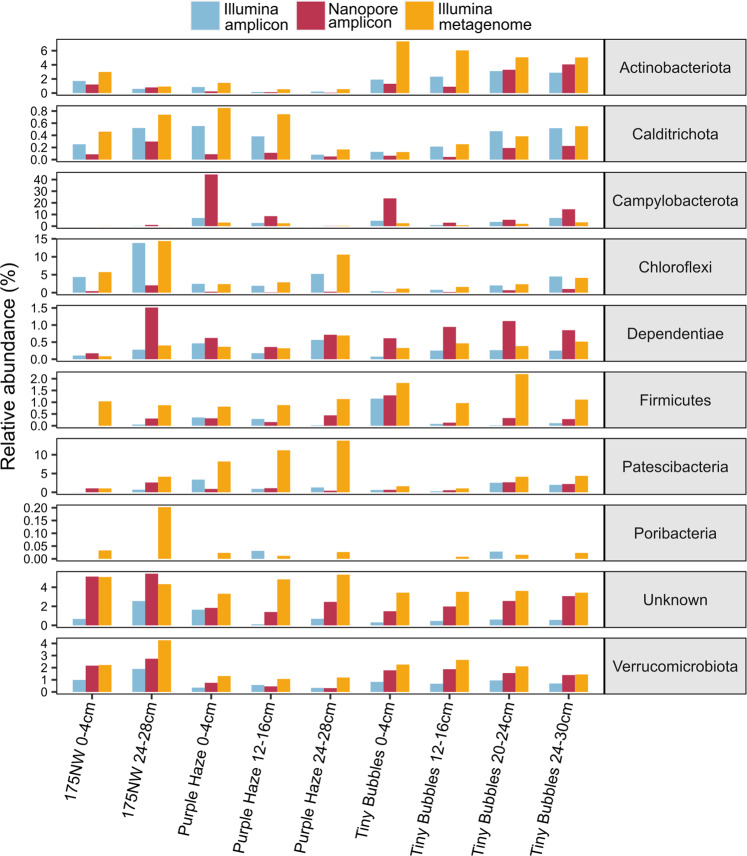
Comparison of relative abundance of a selection of differentially abundant phyla between Nanopore amplicon, Illumina amplicon, and Illumina metagenome data sets. Note the different scales of the *y*-axes. See also Table S3.

To assess how amplicon-based methods compared to primer-free shotgun sequencing, 16S rRNA genes were reconstructed from nine Illumina metagenomes and were compared to the Nanopore and Illumina amplicon libraries. At the phylum level, the Illumina metagenome relative abundances correlated very well with the Nanopore amplicon relative abundances (Pearson’s *r* = 0.876), and almost as high as the correlation between Illumina amplicon and Illumina metagenome relative abundances (Pearson’s *r* = 0.969). Similar to the Nanopore-Illumina amplicon comparison, the phylum Chloroflexi was much more abundant in the Illumina metagenome data set, and the phylum Campylobacterota was more abundant in the Nanopore data set (Fig. 6), suggesting that the Nanopore primers for full-length 16S rRNA genes under- and over-represent these two phyla, respectively. The phylum Patescibacteria was much higher in the Illumina metagenome data set than in either Nanopore or Illumina amplicon libraries (Fig. 6), suggesting that both sets of PCR primers may result in underestimation of this phylum in amplicon libraries [40]. Similarly, Actinobacterota, Firmicutes, and Poribacteria [41] were significantly more abundant in the Illumina metagenome data set than in both Nanopore and Illumina amplicon libraries (Fig. 6), again highlighting how PCR primers can result in underrepresentation or exclusion of some microbial diversity.

A *BLAST* search was performed using the 16S rRNA gene sequences reconstructed from the metagenomes as queries against a database made from the Nanopore amplicon gene sequences. Of the 407 16S rRNA sequences assembled from the metagenomes that were over 800 bp long, 284 (69.8%) had *BLAST* hits with greater than 97% identity to the Nanopore sequences. This shows that the majority of Nanopore sequences, even without error correction, would fall within the traditional 97% identity boundary for operational taxonomic units (OTUs), with near full-length Illumina 16S rRNA gene sequences. There were no *BLAST* matches with 100% identity due to the higher Nanopore error rate.

### User defined database for targeted sequence identification without an internet connection

**Fig. 7:**
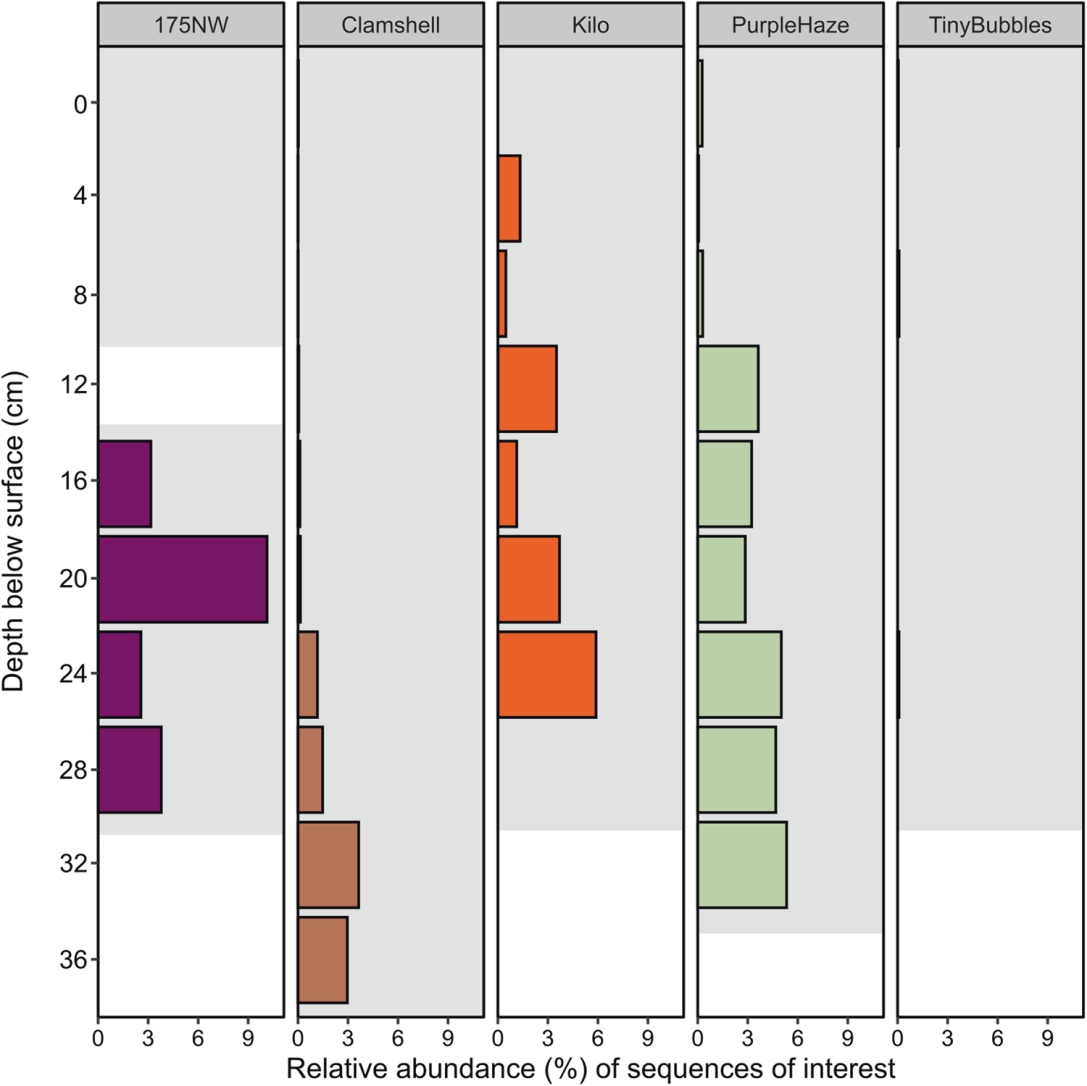
Combined relative abundance and distribution of 21 taxa of interest in each sample. *SituSeq* Stream 2 was used to conduct a *BLAST* search against a custom database of species indicative of hydrocarbon seepage (Data S2). The shaded gray area shows the depths of the samples that were sequenced for each core.

In addition to assigning taxonomy to all Nanopore reads based on Silva version 138.1 (Fig. 3A), the *SituSeq* workflow presented here supports an additional option (Stream 2) to use *BLAST* to match Nanopore amplicon sequences to a pre-populated user-defined database containing sequences of interest. This enables meaningful context-specific data analysis without an internet connection. One of the goals of the research expedition was to identify sediment samples in close proximity to hydrocarbon seepage using the presence of bacterial taxa previously found to be associated with hydrocarbon seeps. Thus, in our case, a database of 21 hydrocarbon-associated bacterial lineages from long cores surrounding deep sea cold seeps was used [27]. Longer cores (up to 10 mbsf) were used to establish these hydrocarbon-associated lineages [27] and thus could potentially skew results here to highlight deeper samples. All 21 of these sequences had *BLAST* hits to Nanopore sequences with >97% identity. Hits were found from a total of 4541 Nanopore sequences in 32 samples, with the sites 175NW and Purple Haze having the highest average relative abundance of hydrocarbon-associated lineages (Fig. 7). Hits at the 175NW site were only found ≥16 cm depth, whereas the relative abundance of the hydrocarbon-associated lineages at the Purple Haze site increased greatly ≥12 cm depth. These results may suggest micro-seepage (i.e., not detected on the ROV footage) occurring at 175NW. The Kilo site also showed high abundance of hydrocarbon-associated species (2.3% on average), while Clamshell site had a higher abundance of hydrocarbon-associated species in deeper samples. Tiny Bubbles site had the fewest hits with only 21 matches across all depths. In this particular case, the Kilo site, as well as deeper samples from 175NW, Purple Haze, and Clamshell sites, were deemed to be of interest for further investigation of hydrocarbon-associated species during the field expedition, whereas the Tiny Bubbles site was de-prioritized for this purpose.

### Subsampling of Nanopore amplicon reads to reduce the time required for the overall workflow

Taxonomic assignment of 16S rRNA gene sequences (Stream 1A) is the computational bottleneck in the *SituSeq* analysis workflow. To speed this up and reduce the amount of time needed for sequencing, a smaller subsampled data set was assessed to see whether fewer sequences would increase computational efficiency without affecting conclusions about microbial community structure and ecology. The community composition of Nanopore libraries subsampled to 1000 sequences was compared to the community composition of the non-subsampled data (average library size of 18,629 sequences).

Ecological trends derived from the 1000 read sequencing depth were very similar to those observed with larger non-subsampled libraries (Table S5). Across all 40 libraries, 61 phyla were identified after subsampling, only 5 fewer than the 66 phyla detected in the larger data set despite removing 705,171 reads (95% of the Nanopore data set). At the genus level, 479 were detected in the non-subsampled data, and 279 were detected after subsampling. The 200 missing genera represented very rare groups, with the most abundant of these representing on average only 0.013% and the aggregate average abundance of dropped genera being 0.0007%. A higher subsampling depth is recommended for *SituSeq* users interested in rare taxa. At the phylum level, the effect of location (site) on community structure was still significant (ANOSIM statistic *R* = 0.382, *p* < 1e-4), as was the relationship between depth and community structure (Mantel statistic r: 0.333, *p* < 1e-4). After subsampling, the same phyla and genera were differentially abundant between locations (Tables S5 and S6, respectively), with the exception of some rare taxa. The same ecological trends were identified in the subsampled data set when assessing beta diversity at the genus level (Fig. S4), demonstrating that as few as 1000 sequences per sample adequately captured the ecological trends for deep sea hydrocarbon seep environments using *SituSeq*.

## Discussion

The ability to rapidly sequence and analyze samples completely offline without an internet connection offers major advantages in settings such as remote field work and rapid point-of-care diagnostics. This study of deep sea sediments showcased the *SituSeq* workflow, demonstrating robust offline analysis of 16S rRNA gene sequences obtained using the highly portable Oxford Nanopore MinION sequencer. The method and interpretations were verified here by comparing *SituSeq* results to standard Illumina MiSeq sequencing of the V4 region of the 16S rRNA gene, and to Illumina NovaSeq metagenome-derived 16S rRNA gene sequences. Overall, there was very high correlation between the methods, with the main discrepancies likely due to preferential amplification by different PCR primer pairs [42–44] rather than being a function of sequencing platform used. *SituSeq* is designed to be simple enough to be implemented by users with little bioinformatics experience as it can be run completely by copying and pasting code into R without any knowledge of command line. The simplicity of the workflow is beneficial for remote deployment where teams of experts can be few in number, yet important decisions, like site selection during environmental surveys or on-site medical diagnoses in resource-poor settings, must be made rapidly and accurately.

Several workflows with varying strategies and goals currently exist for the analysis of Nanopore-sequenced 16S rRNA gene data, as reviewed in detail elsewhere [8, 45]. For example, the *spaghetti* pipeline [46] is designed to aid targeted bioprospecting in the field, which is a similar objective to the present study. The *spaghetti* pipeline comprises multiple steps including removal of primers and adapters with *Porechop* (no longer supported), filtering with *Nanofilt* [47], quality control with *Nanostat* [47], and *minimap2* [48] for mapping long reads to the Silva database. Accordingly, the *spaghetti* workflow depends on installation of multiple separate programs, and *minimap2* for taxonomic assignment, increasing the computing power and bioinformatics expertise required [48]. Recently, Curry et al. (2022) [49] developed *Emu*, a command-line workflow for community profiling of 16S rRNA gene Nanopore sequencing data. *Emu* relies on an expectation-maximization algorithm to correct for the inherent sequencing errors of Nanopore and uses *minimap2* to map long reads to a database. *Emu* produced highly accurate results compared to conventional sequencing methods, but analysis of diverse environmental communities was computationally intensive, requiring more threads and RAM than is usually available on a standard fieldwork laptop. EPI2ME is the standard 16S rRNA analysis and annotation software from Oxford Nanopore and is accessed through a graphical user interface (https://epi2me.nanoporetech.com/). However, it is cloud based and requires an internet connection to use. Due to the ease of offline use and minimal requirements for software installation and computing power, *SituSeq* is a valuable addition to this suite of Nanopore 16S rRNA gene analysis workflows.

Depending on the use-case, mock communities may be available and provide helpful positive controls to combat the lower accuracy of Nanopore sequencing. However, this is not the case for the largely uncharacterized microbial diversity in deep sea sediments. In the absence of such controls, the findings of other studies that have analyzed known samples with Nanopore and alternatives should be referenced [28, 49, 50]. In the present study, the uncultured and unclassified nature of important taxa resident in deep sea sediments was overcome by using a customized database of indicator sequences derived from other seabed cold seep sites [27, 29]. In other uses of *SituSeq*, well-designed local databases queried from a standard laptop can similarly offer an important strategy for rapid identification of environmental or medical taxa of interest. Rapid diagnostic approaches such as isothermal PCR reactions that rely on specific primers instead of sequencing may reliably identify a given bacterial pathogen [51, 52]. However, in the case of a negative result, those assays would need to be repeated with different primers targeting other specific pathogens to achieve a diagnosis. *SituSeq* with a well-designed database would overcome this, and potentially diagnose mysterious cases in remote settings [53].

The ability to characterize a microbiome in real-time could greatly aid many fieldwork expeditions and help researchers make informed decisions about which samples to focus on. For instance, real-time results identifying taxa of interest would aid in the selection of samples for more in-depth analysis requiring more material (e.g., metagenomics, metaproteomics). Studies requiring enrichment or incubation from environmental samples would also benefit from knowing the contents of the inoculant beforehand, and methods like *SituSeq* could be used to target samples containing coveted species for cultivation [46]. In addition, the ability to sequence at source could potentially reduce the number of samples needing to be stored and transported (reducing the cost and risks associated with these tasks), could aid in the characterization of sensitive samples [54], or could be used to characterize microbial community shifts taking place in real time. Using the Nanopore MinION sequencer with the Flongle adapter is relatively inexpensive, as the costs per sample are approximately $30 USD with additional cost savings possible depending on chosen consumables (Table S7). Upfront costs for Nanopore sequencing equipment are roughly $4500 USD, which is more than an order of magnitude cheaper than the conventionally used Illumina MiSeq instrument. The relative accessibility of Nanopore sequencing, matched here with the easy-to-use *SituSeq* workflow, constitutes a step towards democratizing genomics [45, 55].

The *SituSeq* workflow presented here is designed to produce rapid results that can be accessed in the field. The workflow results are highly comparable to what is provided by a conventional 16S rRNA gene amplicon analysis of a variable region using second generation sequencing technologies. Portable Nanopore sequencing in combination with easy and reliable workflows, like the one presented here, will expand the accessibility of sequencing beyond previous technological and economic limits.

## Supplementary information

Supplementary Material.

Supplementary Data 1.

Supplementary Data 2.

Supplementary Data 3.

Supplementary Data 4.

## Data Availability

All raw sequences used in this study have been deposited in the NCBI BioProject database with accession code PRJNA875933. Illumina 16S rRNA gene amplicon BioSamples: SAMN30633139-SAMN30633178. Nanopore 16S rRNA gene amplicon BioSamples: SAMN30633887-SAMN30633926. Illumina metagenomes were submitted to the BioSamples: SAMN30647025-SAMN30647033.
