# Supplementary material for: SituSeq: an offline protocol for rapid and remote Nanopore 16S rRNA amplicon sequence analysis
Source: ISME Commun. 2023 Apr 20;3:33. doi: 10.1038/s43705-023-00239-3 (PMC10119094; doi:10.1038/s43705-023-00239-3)
Supplement: Supplementary file 1 — Supplementary Material. [file 43705_2023_239_MOESM1_ESM.pdf]

Supplementary Material

SituSeq: An offline protocol for rapid and remote Nanopore 16S rRNA amplicon sequence analysis

Jackie Zorz, Carmen Li, Anirban Chakraborty, Daniel Gittins, Taylor Surcon, Natasha Morrison, Robbie Bennett, Adam MacDonald, Casey R.J. Hubert

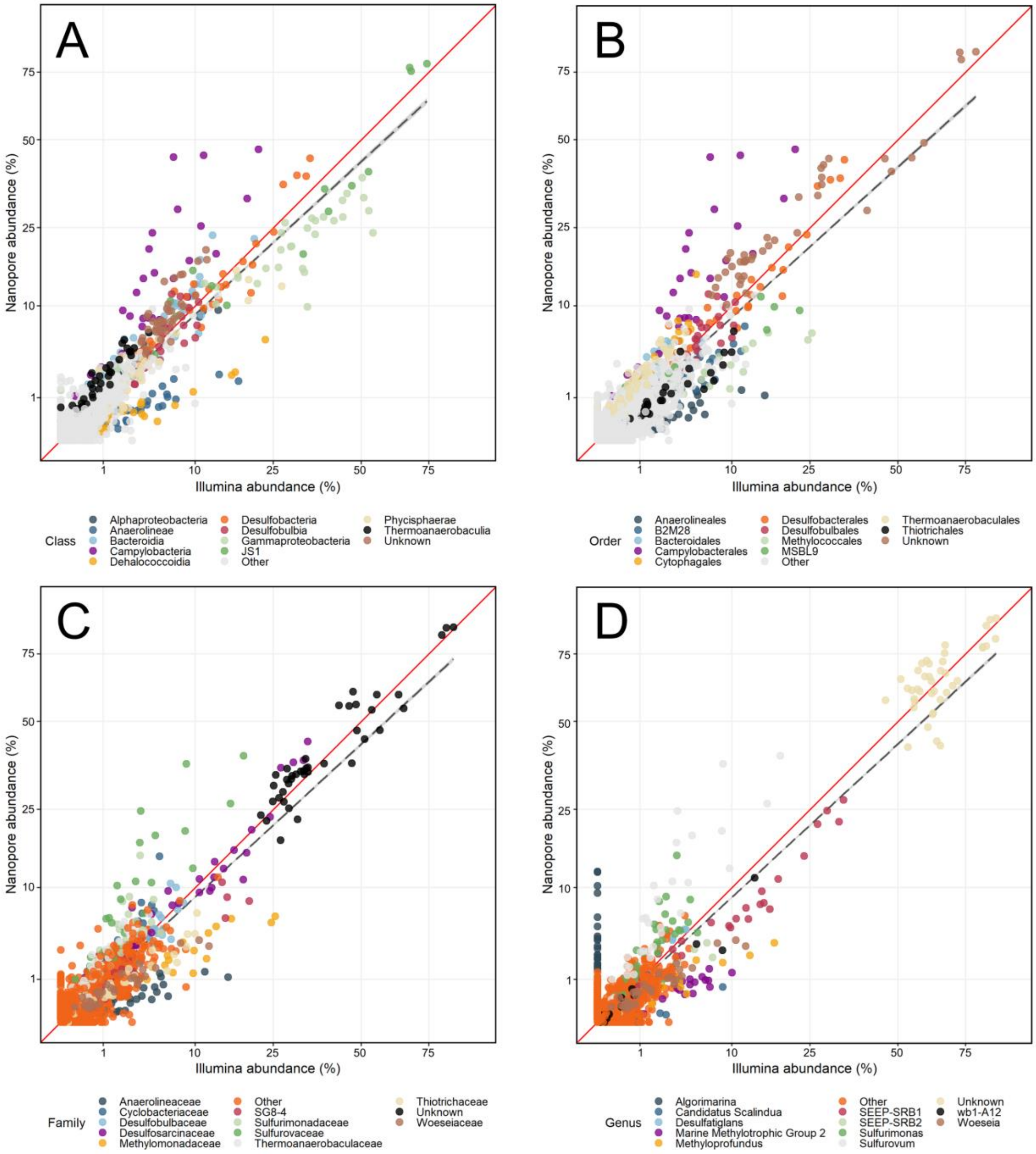

S1 Figure. Correlation between relative abundance of most abundant taxa from Nanopore and Illumina data sets. A) Class level (Pearson's r: 0.902), B) Order level (Pearson's r: 0.914), C) Family level (Pearson's r: 0.958), D) Genus level (Pearson's r: 0.976). "Unknown" refers to the proportion of taxa that were unclassified at that taxonomic level, while "Other" refers to taxa with lower abundance. Red line corresponds to a 1:1 ratio, dashed line shows the linear relationship between the two sequencing methods.

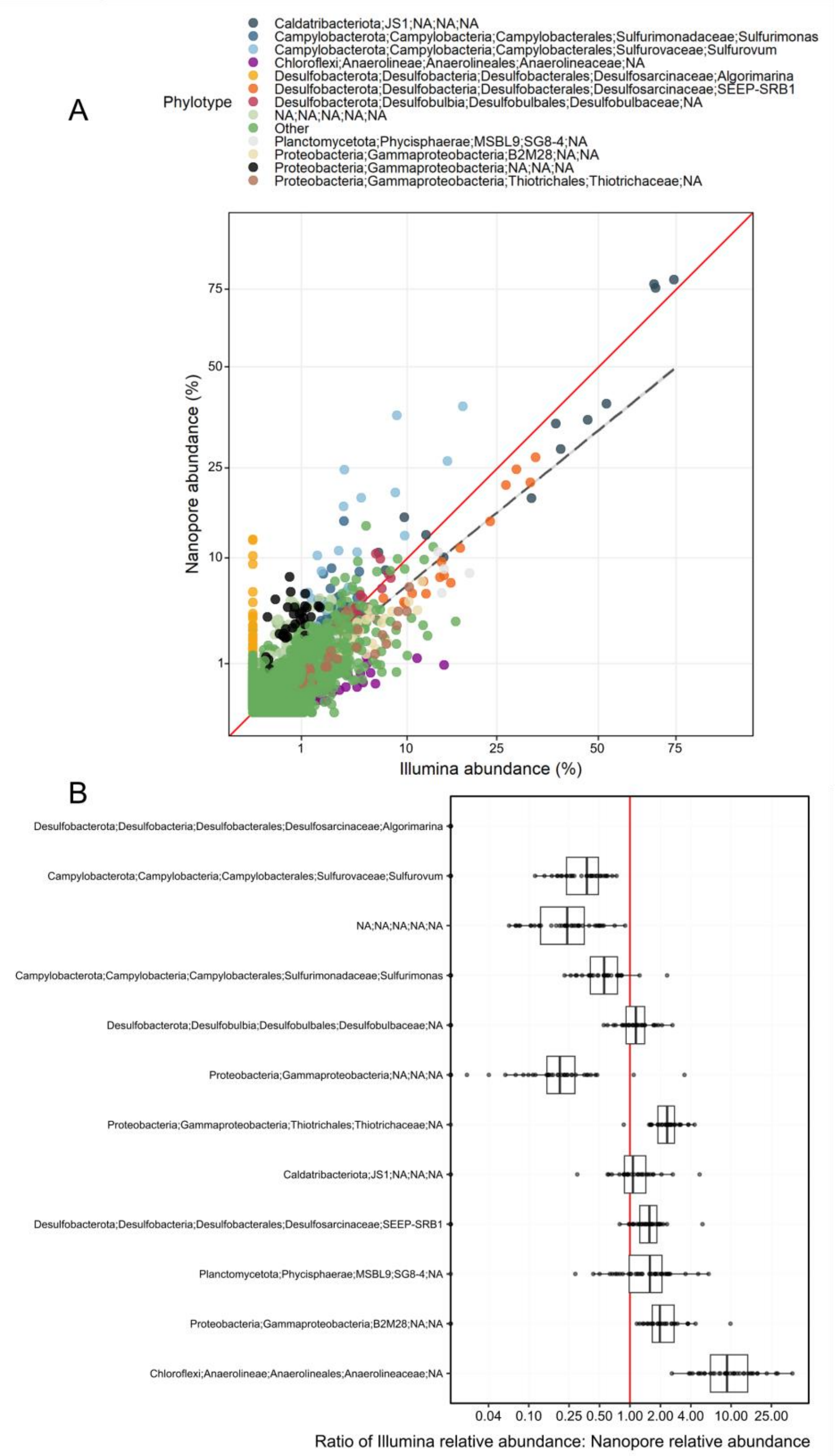

S2 Figure. **Comparison of Nanopore and Illumina amplicon data sets at the level of the highest classified taxonomy, or phylotype**. A) Correlation between Nanopore and Illumina abundance (Pearson’s r: 0.89). B) Ratio of relative abundance between Illumina and Nanopore libraries from most abundant phylotypes.

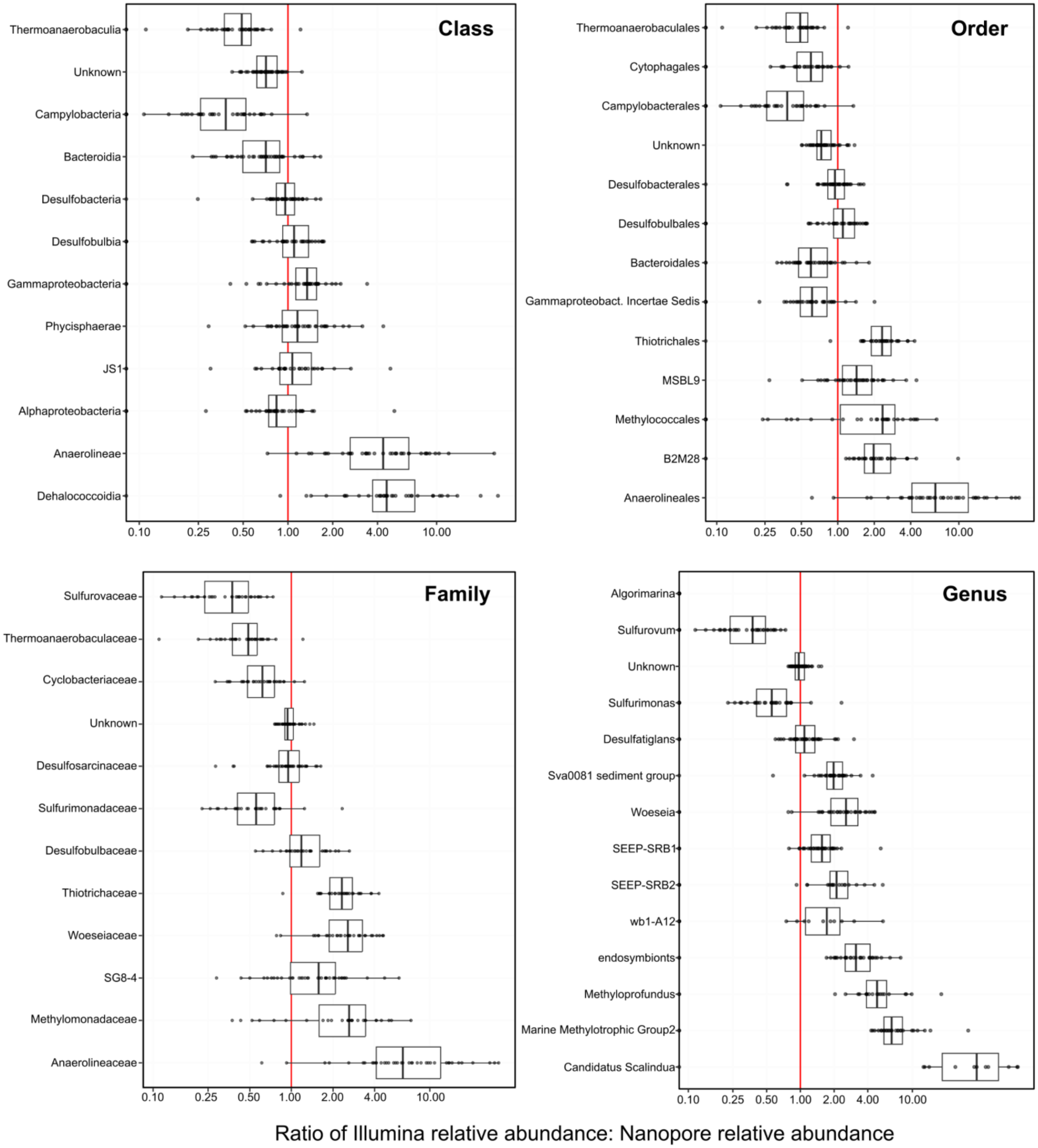

S3 Figure. **Ratio of Illumina abundance to Nanopore abundance of the most abundant taxa at each taxonomic level**. “Unknown” refers to the sequences that were unclassified at each taxonomic level.

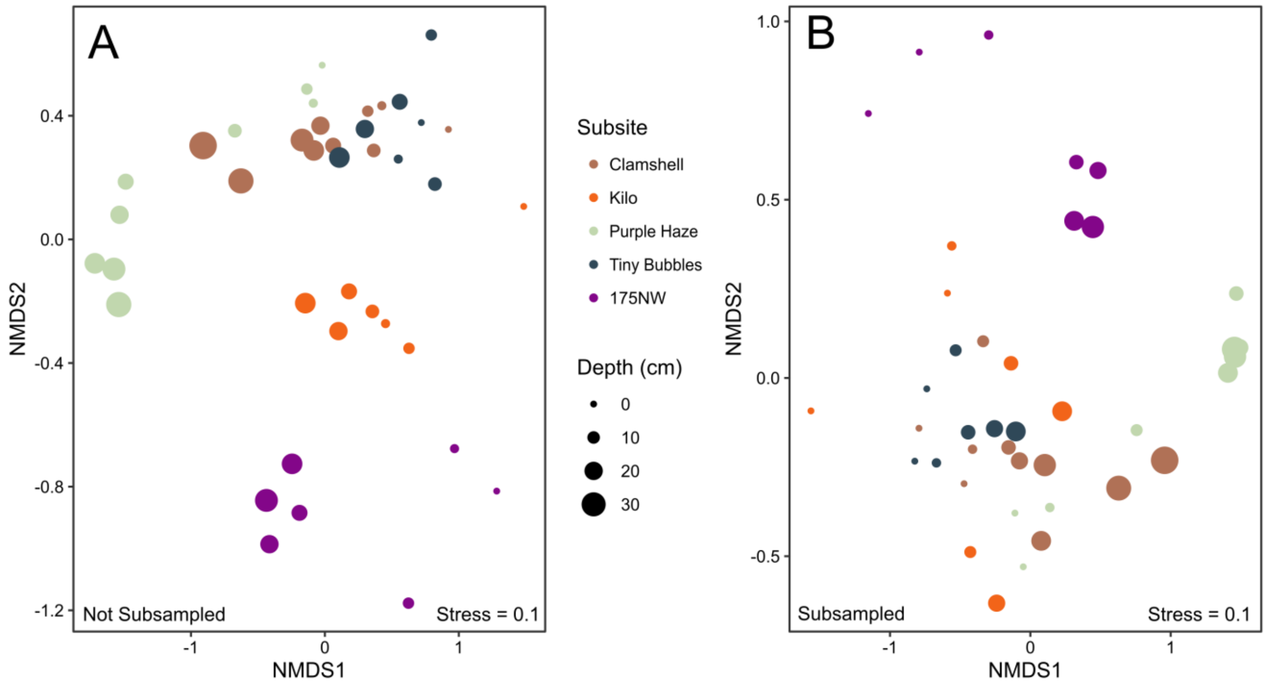

S4 Figure. **Genus level NMDS plot based on Bray-Curtis dissimilarity of the Nanopore data.** (A) Libraries not subsampled, and (B) libraries subsampled to 1000 sequences. Points are coloured based on subsite and sized based on depth below sediment surface. Non-subsampled ANOSIM R (site): 0.52 ($p < 1e-4$); subsampled ANOSIM R (site): 0.43 ($p < 1e-4$)

S1 Table. Primer sequences used for Nanopore and Illumina PCR reactions.

Primer Set	Primer Name	16S rRNA region	Sequence
**Nanopore**	27F/1492R	V1-V9	5’ AGAGTTTGATCMTGGCTCAG 3’<br>5’ CGGTTACCTTGTTACGACTT 3’
**Illumina**	515F/806R	V4	5’ GTGYCAGCMGCCGCGGTAA 3’<br>5’ GGACTACNVGGGTWTCTAAT 3’

S2 Table. Thermocycler programs for both Nanopore and Illumina PCR reactions.

Program	Temperature (°C)	Time (seconds)	Cycles
**Nanopore 16S**	95	60	1
	95	30	25
	55	45	
	72	120	
	72	300	1
**Illumina 16S**	95	180	1
	95	30	10
	60	45	
	72	60	
	95	30	20
	55	45	
	72	60	
	72	300	1
**Illumina Index**	95	180	1
	95	30	10
	55	45	
	72	60	
	72	300	1

S3 Table. Differentially abundant phyla identified for various groupings. Calculated by the *multipatt* function in the package *indicspecies*. Significance codes: 0*** ; 0.001** ; 0.01*

Test	Group	Phylum	Score
**Nanopore amplicon vs Illumina amplicon vs Illumina Metagenome (all sequences) (n=27)**	Nanopore amplicon	Dependentiae	0.625**
		Campylobacterota	0.458*
	Illumina metagenome	Firmicutes	0.739***
		RCP2-54	0.639***
		Patescibacteria	0.602**
		Synergistota	0.597**
		WPS-2	0.585*
		Aquificota	0.580*
		Halanaerobiaeota	0.511*
		NKB15	0.475*
		Deferrisomatota	0.464*
		Actinobacteriota	0.459*
		Poribacteria	0.427**
		AncK6	0.342*
	Illumina amplicon + Illumina metagenome	Calditrichota	0.575**
		Chloroflexi	0.481*
	Nanopore amplicon + Illumina metagenome	Unknown	0.734***
		Verrucomicrobiota	0.492*
		Margulisbacteria	0.480*
**Nanopore amplicon vs Illumina amplicon (all sequences) (n=80)**	Nanopore amplicon	Unknown	0.741***
		Dependentiae	0.618***
		Margulisbacteria	0.386***
		Verrucomicrobiota	0.368***
		Campylobacterota	0.333***
		Patescibacteria	0.311**
		MAT-CR-M4-B07	0.290**
		Deferrisomatota	0.285**
		Modulibacteria	0.244*
	Illumina amplicon	Chloroflexi	0.438***
		Calditrichota	0.362***
		Poribacteria	0.329***
		NKB15	0.256**
		Marinimicrobia (SAR406)	0.241*
		Proteobacteria	0.232*
		Halanaerobiaeota	0.190***
**Location – Nanopore samples (all sequences) (n=40)**	Purple Haze	WS2	0.770***
		Caldatribacteriota	0.636**
		Acetothermia	0.586***
		Caldisericota	0.535**
		FCPU426	0.512*
		Cloacimonadota	0.511**
		Desulfobacterota	0.478*
		Fermentibacterota	0.421*
		Desantisbacteria	0.416*
	175NW	Unknown	0.907***
		Sumerlaeota	0.875***
		Acidobacteriota	0.849***
		Planctomycetota	0.795***
		Armatimonadota	0.679***
		Verrucomicrobiota	0.672***
		Nitrospinota	0.672***
		BH180-139	0.644**
		Hydrogenedentes	0.624***
		10bav-F6	0.606**

		GN01	0.596***
		WOR-1	0.587**
		Aerophobota	0.555**
		Chloroflexi	0.549**
		Nitrospirota	0.538**
		Methylomirabilota	0.532***
		NB1-j	0.505*
		Elusimicrobiota	0.499**
		Dadabacteria	0.496**
		SAR324 clade	0.492***
		Gemmatimonadota	0.490*
		Marinimicrobia (SAR406)	0.452*
		AncK6	0.442***
		MBNT15	0.440*
		Zixibacteria	0.436*
		Patescibacteria	0.407*
	Tiny Bubbles + Clamshell	Actinobacteriota	0.597**
		Bacteroidota	0.512**
	Kilo	Schekmanbacteria	0.725***
		Deferrisomatota	0.678***
		Modulibacteria	0.665***
		Campylobacterota	0.604**
		DTB120	0.550**
**Location – Illumina samples (all sequences) (n = 40)**	Purple Haze	WS2	0.808***
		Acetothermia	0.708***
		Cloacimonadota	0.706***
		Caldatribacteriota	0.681***
		CK-2C2-2	0.667***
		Dependentiae	0.643***
		Fermentibacterota	0.554**
		Patescibacteria	0.553**
		Desantisbacteria	0.521*
		Caldisericota	0.505*
		Desulfobacterota	0.486**
		TA06	0.485*
		FCPU426	0.478*
		Marinimicrobia (SAR406)	0.405*
	175NW	Acidobacteriota	0.917***
		Planctomycetota	0.828***
		Armatimonadota	0.670***
		Aerophobota	0.664***
		Unknown	0.643***
		Sumerlaeota	0.616**
		Methylomirabilota	0.603***
		10bav-F6	0.578**
		BH180-139	0.563**
		Nitrospinota	0.561**
		Nitrospirota	0.559**
		Chlorovlexi	0.550**
		Dadabacteria	0.525**
		WOR-1	0.495*
		SAR324 clade	0.493**
		Hydrogenedentes	0.492*
		NB1-j	0.492*
		MBNT15	0.485*
		Gemmatimonadota	0.425*
		Verrucomicrobiota	0.397*
		Actinobacteriota	0.713***

	Tiny Bubbles + Clamshell	Bacteroidota	0.437*
		Myxococcota	0.406*
	Kilo	Deferrisomatota	0.777***
		Campylobacterota	0.689***
		DTB120	0.676***
		Poribacteria	0.664***
		Fibrobacterota	0.577**
		NKB15	0.571**
		Schekmanbacteria	0.534**
		Margulisbacteria	0.431*

S4 Table. ANOSIM and Mantel statistics calculated with Bray-Curtis dissimilarity for Illumina and Nanopore data sets combined at various taxonomic levels.

Taxonomic Level	ANOSIM – Sequencing Technology (*p* value)	ANOSIM – Site (*p* value)	Mantel – Depth (*p* value)
Phylum	0.06 (0.008)	0.38 (< $1e^{-4}$)	0.33 (< $1e^{-4}$)
Class	0.05 (0.02)	0.41 (< $1e^{-4}$)	0.34 (< $1e^{-4}$)
Order	0.07 (0.006)	0.50 (< $1e^{-4}$)	0.29 (< $1e^{-4}$)
Family	0.06 (0.01)	0.53 (< $1e^{-4}$)	0.28 (< $1e^{-4}$)
Genus	0.12 (< $1e^{-4}$)	0.44 (< $1e^{-4}$)	0.22 (< $1e^{-4}$)
Phylotype*	0.13 (< $1e^{-4}$)	0.49 (< $1e^{-4}$)	0.35 (< $1e^{-4}$)

*highest taxonomic classification

S5 Table. Differentially abundant phyla identified for various groupings. Calculated by the *multipatt* function in the package *indicspecies* with the number of reads per sample subsampled to 1000. Significance codes: 0*** ; 0.001** ; 0.01*

Test	Group	Phylum	Score
**Location – Nanopore samples (1000 sequences per sample) (n = 40)**	Purple Haze	Caldatribacteriota	0.635***
		WS2	0.612***
		Acetothermia	0.536**
		Cloacimonadota	0.485*
		Desulfobacterota	0.481*
		Caldisericota	0.450**
	175NW	Unknown	0.854***
		Sumerlaeota	0.841***
		Acidobacteriota	0.831***
		Planctomycetota	0.810***
		Nitrospinota	0.697***
		Armatimonadota	0.642***
		Verrucomicrobiota	0.615***
		BH180-139	0.588**
		Hydrogenedentes	0.568***
		Chloroflexi	0.559**
		10bav-F6	0.550**
		Elusimcrobiota	0.525**
		Methylomirabilota	0.507***
		Nitrospirota	0.496*
		NB1-j	0.493*
		Gemmatimonadota	0.493*
		WOR-1	0.489**
		Aerophobota	0.480**
		Bdellovibrionota	0.476*
		SAR324 clade	0.475***
	Tiny Bubbles + Clamshell	Actinobacteriota	0.62***
		Bacteroidota	0.54**
	Kilo	Schekmanbacteria	0.705***
		Campylobacterota	0.619**
		DTB120	0.517*
		Deferrisomatota	0.438*
**Location – Illumina samples (1000 sequences per sample) (n = 40)**	Purple Haze	WS2	0.687***
		Cloacimonadota	0.682***
		Caldatribacteriota	0.682***
		Acetothermia	0.673***
		Dependentiae	0.557***
		CK-2C2-2	0.540**
		Patescibacteria	0.538**
		Desulfobacterota	0.472*
		Fermentibacterota	0.459*
		TA06	0.452*
	175NW	Acidobacteriota	0.910***
		Planctomycetota	0.811***
		Methylomirabilota	0.599***
		Nitrospirota	0.593**
		Aerophobota	0.583**
		Armatimonadota	0.583**
		10bav-F6	0.559**
		Chloroflexi	0.538**
		Nitrospinota	0.527**

		Unknown	0.515*
		NB1-j	0.492*
		Dadabacteria	0.468*
		SAR324 clade	0.449**
		WOR-1	0.438*
	Tiny Bubbles + Clamshell	Actinobacteriota	0.670***
		Myxococcota	0.474*
		Bacteroidota	0.434*
	Kilo	Campylobacterota	0.686***
		Fibrobacterota	0.629**
		Schekmanbacteria	0.609**

S6 Table. Significantly differentially abundant genera identified for various groupings within the Nanopore data. Calculated by the *multipatt* function in the package *indicspecies* with either no subsampling or libraries subsampled to 1000 reads. Only showing highly significant results, $p < 0.001$***

Test	Group	Genus	Score
**Location – Nanopore samples (all sequences) (n = 40)**	Purple Haze	SEEP-SRB1	0.678***
	175NW	Candidatus Omnitrophus	0.895***
		Methyloligella	0.866***
		Sumerlaea	0.860***
		SM23-31	0.780***
		Bythopirellula	0.664***
		Spirochaeta	0.631***
		Filomicrobium	0.616***
		P3OB-42	0.610***
		Limibacillus	0.556***
		Wb1-A12	0.532***
	Tiny Bubbles + Clamshell	Pltb-vmat-59	0.775***
		Actibacter	0.765***
		Defluviicoccus	0.751***
		Desulfobulbus	0.713***
		Subgroup 23	0.703***
		IheB2-23	0.695***
		TPD-58	0.642***
		Marinimicrobium	0.636***
	Kilo	MSBL7	0.816***
		Calorithrix	0.768***
		Prolixibacter	0.716***
		Candidatus Thiodiazotropha	0.687***
		Sulfurovum	0.681***
		Deferrisoma	0.678***
		Candidatus Thiopilula	0.666***
		Desulfocapsa	0.585***
		Marinosulfonomonas	0.575***
		Chitinivibrio	0.548***
		Pseudozobellia	0.542***
		Mangrovibacterium	0.510***
**Location – Nanopore samples (1000 sequences per sample) (n = 40)**	Purple Haze	SEEP-SRB1	0.674***
	175NW	Candidatus Omnitrophus	0.802***
		Methyloligella	0.747***
		Sumerlaea	0.680***
		Wb1-A12	0.548***
	Tiny Bubbles + Clamshell	Defluviicoccus	0.745***
		Subgroup 23	0.730***
		Marinimicrobium	0.692***
		Marine Methylotrophic Group 2	0.663***
	Kilo	MSBL7	0.715***
		Candidatus Thiodiazotropha	0.679***
		Sulfurovum	0.678***
		Deferrisoma	0.625***
		Fusibacter	0.468***

S7 Table. Example of costs associated with 16S rRNA *in situ* sequencing using a Nanopore MinION and Flongle adapter and the *SituSeq* workflow.

Item	Company	Cost (USD)	Cost per sample (USD)
DNA Extraction			
DNA extraction kit<br>DNeasy PowerLyzer PowerSoil kit	Qiagen	396	8
DNA polymerase<br>KAPA HiFi HotStart master mix	Roche	750	6
Nanopore Sequencing			
minION	Nanopore	1000	
minIT*/good laptop^	Nanopore	2000	
Flongle flowcell	Nanopore	90	7.5 (12 samples)
Flongle adapter starter pack<br>(contains adapter + 12 flowcells)	Nanopore	1460	
16S Barcoding kit<br>(12 barcodes, 6 reactions)	Nanopore	650	~9
Totals			
Cost for consumables (per sample)**	30.5		
Costs for equipment**	4460		

*minIT has been discontinued
^a laptop with good specs (e.g. Dell Inspiron 13-7378 laptop with 16 GB RAM and 512 GB SSD)
**Costs do not include consumables like pipette tips, tubes, etc., or other lab equipment like centrifuges and PCR machines. See Edwards et al. (2022) or Werner et al. (2022) for further information on detailed packing lists and tips for *in situ* sequencing in the field.

Supplementary Data (separate files):

S1 Data. *SituSeq* code

S2 Data. Sequences for species of interest BLAST search

S3 Data. Abundance of taxonomic phylotypes assigned to Nanopore sequences across samples

S4 Data. Abundance of taxonomic phylotypes assigned to Illumina sequences across samples
